# Does lucency equate to revision? A five-year retrospective review of Attune and Triathlon total knee arthroplasty

**DOI:** 10.1007/s00167-023-07509-6

**Published:** 2023-07-30

**Authors:** Paul O’Donovan, Timothy McAleese, James Harty

**Affiliations:** 1 School of Medicine, University College Cork, Brookfield Health Sciences Complex, College Road, Cork, Ireland; 2 Department of Trauma and Orthopaedics, South Infirmary Victoria University Hospital, Cork, Ireland

**Keywords:** Total knee replacement, Radiolucent lines, Aseptic loosening, Tibial component, Implant–cement interface, Attune^®^, Triathlon^®^

## Abstract

**Purpose:**

The Attune^®^ total knee arthroplasty system was introduced in 2013 to address lingering issues of patient dissatisfaction. However, recent literature reports concerns of early tibial tray debonding. The aim of this study was to compare the incidence of radiolucent lines, survivorship and patient reported outcome-measures between the Attune^®^ system and the well-established Triathlon^®^ system.

**Methods:**

This retrospective database review was conducted at a single institution in Cork, Ireland. All primary Attune^®^ (*N* = 445) and Triathlon^®^ (*N* = 285) systems implanted between 2015 and 2016 were reviewed. Radiolucent lines were assessed for those with a minimum two-year radiological follow-up (Attune^®^ = 338; Triathlon^®^ = 231). X-rays were taken post op, at 6 months, 2 years and 5 years. Radiolucent lines were documented using the Modern Knee Society Radiographic System. Five-year survival was assessed using Kaplan–Meier analysis with the Log Rank method to determine statistical significance. The Oxford Knee Score and EQ-5D-5L, were collected pre-op, at 6 months, 2 years and 5 years post-operatively and compared using the Kruskal–Wallis Test.

**Results:**

The Attune^®^ had a higher proportion of radiolucent lines at the tibial tray [87.1% (54/62) vs 61.4% (27/44); *p* = 0.001] and at the implant–cement interface [62.9% (39/62) vs 43.2% (19/44); *p* = 0.02]. Conversely, the Triathlon^®^ had a higher proportion AT the femur [38.6% (17/44) vs 12.9% (8/62); *p* = 0.001] and at the cement–bone interface [56.8% (25/44) vs 37.1% (23/62); *p* = 0.02]. The overall frequency of radiolucent lines was similar in both the Attune^®^ and Triathlon^®^ groups [17.8%, (60/338) vs 17.7%, (41/231); *p* = 0.49]. There was no difference in revision-free survival analysis at 5 years (Attune^®^ 97.8% vs Triathlon^®^ 95.8%; *p* = 0.129). The Attune^®^ performed better at 5 years in the Oxford Knee Score [Attune^®^ = 42.6 (SD 5.2) vs Triathlon^®^ = 41 (SD 6.4); *p* = 0.001] and in the EQ-5D [Attune^®^ = 0.773 (SD 0.187) vs Triathlon^®^ = 0.729 (SD 0.218); *p* = 0.013]. There was no difference at 5 years in the EQ-VAS [Attune^®^ = 80.4 (SD 13.7) vs Triathlon^®^ = 78.5 (SD 15.3); *p* = 0.25].

**Conclusion:**

The Attune^®^ system exhibited a higher incidence of  radiolucent lines at the tibial tray. However, this did not lead to decreased survivorship at medium term follow-up compared to the Triathlon^®^. Furthermore, improvements in patient reported outcomes modestly favoured the Attune^®^ system.

**Level of evidence:**

III.

## Introduction

Total knee arthroplasty (TKA) has been shown to deliver pain relief and restore function for patients with end-stage joint disease [1]. However, several studies have reported that up to 10–20% of patients remain dissatisfied after the procedure [5]. With the growing market for total knee replacement, implant manufacturers are incentivised to produce more effective implant systems that survive longer and improve patient satisfaction.

The Attune^®^ system *(DePuy Synthes, Warsaw, IN, USA)* was introduced in 2013 to address the residual dissatisfaction rates associated with TKA. It was designed to increase conformity between the femoral component and polyethylene insert with a gradually reducing femoral radius. The design also included an s-curve design of the cam, improved patellofemoral tracking and a new antioxidant polyethylene insert [31, 33]. UK and Australian Registry data reports that the early survival rates of the Attune^®^ system are better than average and excellent [24]. However, concerns have been raised regarding early aseptic loosening at the tibial implant–cement interface [4, 7, 19, 29]. Additionally, other studies have noted an increased occurrence of radiolucent lines (RLLs) using this implant, although the specificity of RLLs for predicting loosening in the Attune^®^ prosthesis remains a cause of contention [10, 11, 17]. Given this concern, the manufacturer redesigned the tibial tray and introduced the Attune^®^ S + in 2017 which featured additional cement pockets with an under cut and increased surface roughness.

Previous research analysing the survivorship and clinical outcomes of the Attune^®^ has acknowledged the lack of non-registry, single centre studies using this implant. These studies have also highlighted the need to further evaluate the Attune^®^ with larger sample sizes and after a longer follow-up period, beyond 2 years [12, 29, 32].

This study presents the largest, single centre case-series evaluating the Attune system at 5-years follow-up. Our primary objective was to assess the occurrence of radiolucent lines, aseptic loosening and survivorship of the Attune^®^ and Triathlon^®^ systems. We specifically wanted to examine our cohort for signs of premature tibial tray loosening. Our secondary objective was to compare these implant systems with regards to patient-reported, functional and overall quality of life outcomes. Our hypothesis is there is no difference between the Attune^®^ and Triathlon^®^ systems in terms of these outcome measures at mid-term follow-up.

## Methods

This study was conducted and reported in keeping with the STROBE guidance for observational studies [31]. We included all primary TKAs using either the Attune^®^ or Triathlon^®^ systems (Total *n* = 730; 445 Attune^®^ and 285 Triathlon^®^) at our hospital over a 2 year period between January 2015 and December 2016. We included cruciate retaining, posterior stabilised, fixed bearing and rotating platform designs. This data is maintained by a local hospital registry as well as the Irish National Orthopaedic Registry (INOR) which was established in 2014.

At our Institution, the Attune^®^ was implanted by two surgeons and the Triathlon^®^ was implanted by three surgeons. All operations were performed with the patient under spinal or general anaesthesia, using a medial parapatellar approach, capsulotomy and patellar eversion. Femoral and tibial resection was performed with a modified measured resection technique. All implants were fully cemented using a third generation technique and high viscosity cement (Palacos R + G, *Heraeus Medical, Wehrheim, Germany*). All patients followed a standardised recovery protocol and post-operative follow-up.

**Fig. 1:**
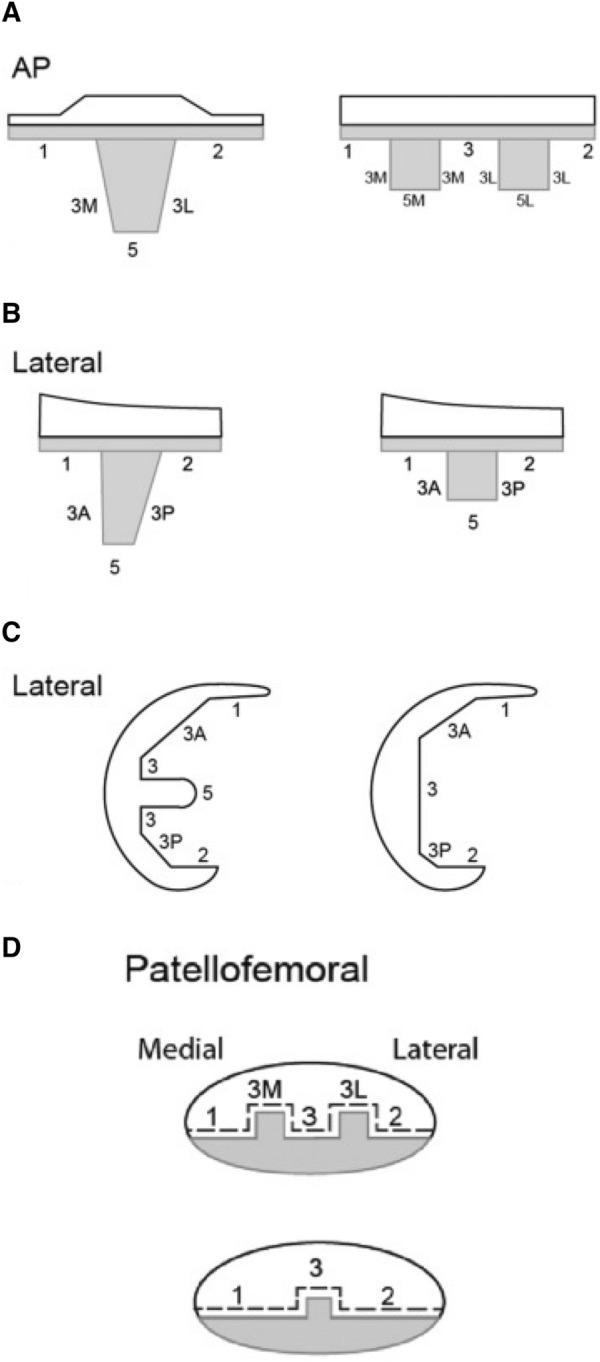
Modern Knee Society radiographic evaluation system (**A**) Coronal and (**B**) sagittal radiographic schematic of keeled and two-peg implants with zones for documentation of radiolucent lines and osteolysis. (**C**) Sagittal plane radiographic schematic of femoral implant with zones denoted for documentation radiolucent lines and osteolysis. (**D**) Patellofemoral view radiographic schematic of multi- or single- peg patella implant with zones denoted for documentation radiolucent lines and osteolysis. Radiolucent lines were documented as “partial” or “complete” and whether they occured at the implant-cement (IC) or cement-bone (CB) interface. (Meneghini et al. [20])

Patients were evaluated prospectively at regular intervals during the 5-year follow-up period. As part of routine follow-up by the national joint registry, anteroposterior (AP) and lateral radiographs of the operative side are performed at 6 months, 2 years and 5 years post-operatively. Evaluation of radiolucent lines (RLL) was performed by 2 independent researchers (PO’D, TM) using the modern knee society radiographic evaluation system (MKSRES) [20]. Each radiograph was reviewed without knowledge of whether the patients had undergone revision for loosening. This blinding process ensured that the assessors bias was minimised. Both assessors were familiar with the existing literature on RLL subtypes and the evaluation of tibial RLLs. RLLs were defined as either between the implant-cement (I/C) or the cement-bone (C/B) interface, where lucent lines in specific zones were categorised as partial or complete and stable or progressive [20]. Previous studies have shown that the MKSRES tool has demonstrated high rates of inter-observer and intra-observer reliability [2] (Fig. 1).

Patient reported outcome measures (PROMs) were collected pre-operatively and then at 6 months, 2 years and 5 years post-operatively. The Oxford Knee Score (OKS) was used for all patients. This measure consists of 12 questions evaluating knee pain and function over the preceding 4 weeks. Each question is scored on a Likert scale from 1 to 4 and a total score between 0 (worst) and 48 (best) is calculated.

Patient’s general quality of life and health was also analysed using the EQ-5D-5L questionnaire. This was completed by all patients from when it became routinely collected in May 2016. As a result a number of patients did not have the EQ-5D-5L collected pre-operatively or at 6 months but all patients provided this measurement at 2 years and 5 years follow-up. Minimum clinically important differences (MCIDs) were calculated for each PROM using a distribution-based method and were used to define meaningful clinical improvement. The MCID score for the OKS was 6 points [9, 13].

This study was approved by the Clinical Research Ethics Committee of the Cork Teaching Hospitals, University College Cork (IRB Number: ECM 6 (f) 05/05/2020).

### Statistical analysis

Statistical analysis was performed using SPSS 25 (IBM). The level of significance was set at p < 0.05. The PROMs (EQ-5D-5L and OKS) and rate of RLLs for each implant were compared using a 2-proportion *z*-test and the Kruskal-Wallace test. Survival analysis was conducted using a Kaplan Meier survival curve with the Log-Rank (Mantel-Cox) method to test for statistical significance. The end-point for analysis was defined as revision surgery, which encompassed removal of any component for any reason. Descriptive statistics are displayed as means with standard deviation or range for continuous variables.

Given the large sample size in this study, it was sensitive to small effects in the primary outcome measures. An effect size 0.1 would have been detected with an alpha of 0.05 and 87.09% power for the RLLs analysis. An effect size of 0.05 would have been detected with an alpha of 0.05 and 89.44% power in the analysis of survivorship.

## Results

### Radiolucent lines (RLLs)

569 implants (338 Attune^®^; 231 Triathlon^®^) were included in the radiological evaluation as they had follow-up radiographs of 2 years or longer. The Attune^®^ group had a higher percentage of RLLs present at the tibial component 87.1% vs 61.4% (*p* = 0.001) and at the implant–cement interface 62.9% vs 43.2% (*p* = 0.02). Conversely, the Triathlon^®^ group had a higher percentage of RLLs involving the femoral component 38.6% vs 12.9% (*p* = 0.001) and at the cement-bone interface 56.8% vs 37.1% (*p* = 0.02). There was no difference in the overall number of implants with RLLs or in the nature of complete or progressive RLLs between the Attune^®^ and Triathlon^®^ systems respectively (Table 1).

**Table 1:** Summary of the quantity, characteristics and location of radiolucent lines on post-operative radiographs

Summary of radiolucent lines						
		Attune	%	Triathlon	%	*P* value
No. with lines		60/338	17.8	41/231	17.7	0.4996
Total lines		62		44		
Tibia		54/62	87.1	27/44	61.4	**0.001**
Femur		8/62	12.9	17/44	38.6	**0.0011**
Partial		36/62	58.0	29/44	65.9	0.207
Complete		26/62	41.9	15/44	34.1	0.207
IC		39/62	62.9	19/44	43.2	0.0222
CB		23/62	37.1	25/44	56.8	0.0222
Stable		24/62	38.7	19/44	43.2	0.322
Progressive		38/62	61.3	25/44	56.8	0.322

We have highlighted the results that reached statistical significance in bold. The level of significance was set at *P* <0.05 for this study

**Fig. 2:**
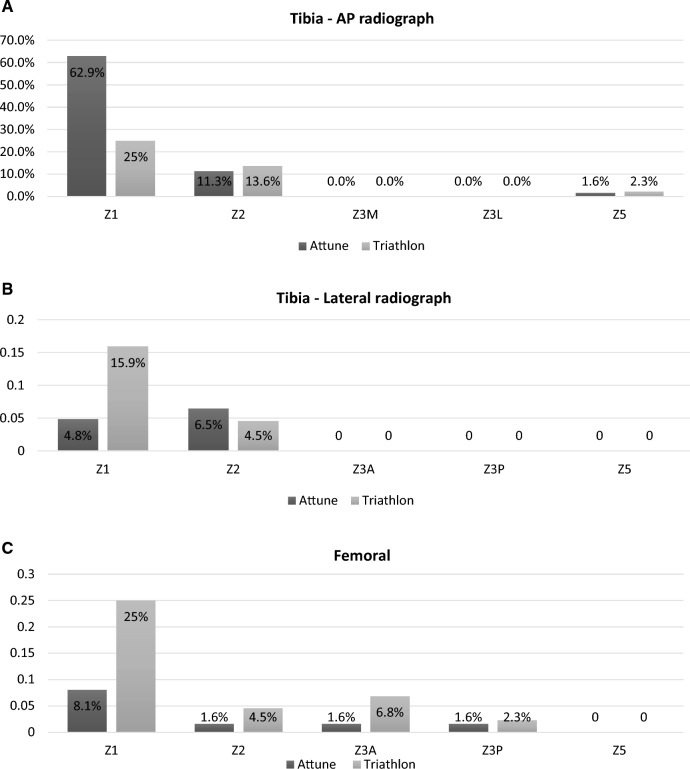
**A** Location of radiolucent Lines (RLLs) for the Attune^®^ and Triathlon^®^ systems on the AP view of the tibial component. Percentages are relative to total RLLs for each system. **B** Location of radiolucent lines (RLLs) for the Attune^®^ and Triathlon^®^ systems on the Tibia – Lateral radiograph of the tibial component. Percentages are relative to total RLLs for each system. **C** Location of radiolucent lines (RLLs) present on the Femoral component for the Attune^®^ and Triathlon^®^ systems. Percentages are relative to total RLLs for each system

For both implants, the majority of RLLs occurred at the medial tibial baseplate on the AP view. In the Attune^®^ group, 62.9% of RLLs occurred at this location while in the Triathlon^®^ group, 25% occurred here and a further 25% were present at the anterior flange of the femoral component (Fig. 2A–C).

### Survivorship

**Fig. 3:**
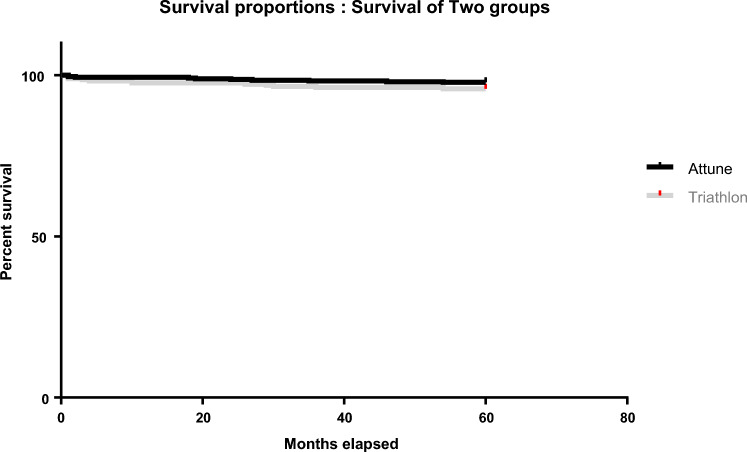
Kaplan Meier survival analysis at 5 years post-operatively. Comparison between the Attune^®^ and Triathlon^®^ systems

Survival analysis showed no statistically significant difference in revision-free survival between the Attune^®^ and Triathlon^®^ systems at 5 years (97.8% vs. 95.8%, *p* = 0.129) (Fig. 3). Similarly, revision rate at 5 years was 2.2% (95% CI 0.9–3.6%) for the Attune^®^ and 4.2% (95% CI 1.9–6.5%) for the Triathlon^®^ (Table 2). Revision was defined as removal of any component for any reason. The reasons for revision and the components revised are outlined in Table 3. All components were revised in 4 cases using the Attune^®^ and 4 cases using the Triathlon^®^. Of these, one patient from each of the Triathlon^®^ and Attune^®^ groups experienced loosening of the tibial component at 30 and 46 months respectively. The 3 remaining Attune^®^ revisions were for instability while the Triathlon^®^ was also revised for 2 cases of instability and 1 case of trauma resulting in a dislocated knee and ruptured extensor mechanism*.*

**Table 2:** 5-year survival analysis for the Attune^®^ and Triathlon^®^ systems.

Survival analysis summary					
Brand	Total N	Revisions	Survived	Percent	95% CI
Attune	445	10	435	97.8%	95.9–98.9
Triathlon	285	12	273	95.8%	92.8–97.8
Overall	730	22	708	97.0%	95.5–98.1

**Table 3:** Rates of revision surgery for all causes sub-categorised by the component revised. There were no tibial components revised in isolation during this study

Brand	Poly	Patella	All components	Infection	Total revised	Total reviewed	%	95% CI
Attune	1	1	4	3	10	445	2.2	0.9–3.6
Triathlon	1	3	4	4	12	285	4.2	1.9–6.5

### Patient reported outcome measures (PROMs)

There were 635 patients with PROMs completed at 5-years follow-up (397 Attune^®^ and 238 Triathlon^®^) (Table 4). The OKS scores for patients in each group were equivalent at 6 months and 2 years follow-up. However, the Attune^®^ scored higher in the OKS at 5 years [Attune^®^, *n* = 398; 42.6 (SD 5.2) vs Triathlon^®^, *n* = 238; 41 (SD 6.4); *p* = 0.001]. It was noted that the pre-operative OKS score was slightly lower in the Triathlon^®^ group so to account for this, the percentage of potential improvement obtained was calculated. This still favoured the Attune^®^ group at 5 years (79.5% vs 75.9%, *p* = 0.005) (Table 4).

**Table 4:** Oxford Knee Scores (OKS) for the Triathlon^®^ and Attune^®^ systems pre-operatively and at 6 months, 2 years and 5 years post-operatively

OKS	Prosthesis	*N*	Mean	Std. deviation	*P*-value
PRE	Attune	416	**20.6**	8.4	**0.001**
	Triathlon	261	**17.8**	8.3	
0.5	Attune	424	**38.7**	6.8	0.162
	Triathlon	263	**37.8**	7.5	
2	Attune	425	**42.6**	5.0	0.105
	Triathlon	261	**41.7**	6.2	
5	Attune	398	**42.6**	5.2	**0.001**
	Triathlon	238	**41.0**	6.4	
5 year improvement	Attune	374	**21.7**	8.7	0.315
	Triathlon	222	**22.5**	8.7	
Potential Improvement	Attune	374	**27.1**	8.1	**0.0005**
	Triathlon	222	**29.6**	8.2	
% attained of potential improvement	Attune	374	**79.5**	0.2	**0.005**
	Triathlon	222	**75.9**	0.2	

EQ-VAS	Prosthesis	*N*	Mean	Std. deviation	*P*-value
PRE	Attune	116	**70.7**	20.6	0.404
	Triathlon	77	**68.7**	19.1	
0.5	Attune	233	**79.9**	13.5	0.659
	Triathlon	159	**84.2**	73.2	
2	Attune	425	**80.9**	15.0	**0.032**
	Triathlon	260	**78.8**	14.7	
5	Attune	397	**80.4**	13.7	0.250
	Triathlon	238	**78.6**	15.3	

We have highlighted the results that reached statistical significance in bold. The level of significance was set at *P* <0.05 for this study

The Attune^®^ performed higher in the EQ-5D-5L at 2 years and at 5 years [0.773 (SD 0.187) vs. 0.729 (SD 0.218); *p* = 0.013]. In the EQ-VAS, the Attune^®^ performed better at 2 years [80.9 (SD 15.03) vs. 78.8 (SD 78.8); *p* = 0.032] although at 5 years the scores were equivalent [Attune^®^, *n* = 397; 80.4 (SD 13.7) vs Triathlon^®^, *n* = 238; 78.6 (SD 15.3); *p* = 0.25] (Table 5).

**Table 5:** A, B EQ-5D-5L (EuroQol five-dimension five-level questionnaire) scores for the Triathlon^®^ and Attune^®^ systems pre-operatively and at 6 months, 2 years and 5 years post-operatively

EQ-5D	Prosthesis	*N*	Mean	Std. deviation	*P*-value
PRE	Attune	116	**0.476**	0.215	0.070
	Triathlon	77	**0.411**	0.249	
0.5	Attune	233	**0.774**	0.175	0.695
	Triathlon	159	**0.765**	0.175	
2	Attune	425	**0.772**	0.197	**0.001**
	Triathlon	261	**0.719**	0.234	
5	Attune	397	**0.773**	0.187	**0.013**
	Triathlon	238	**0.729**	0.218	

We have highlighted the results that reached statistical significance in bold. The level of significance was set at *P* <0.05 for this study

## Discussion

The most important finding of this study was that the Attune® system was associated with a higher incidence of RLLs, particularly under the medial aspect of the tibial baseplate at the I/C interface. However, this did not correlate with an increased rate of implant failure or revision for aseptic loosening. Furthermore, survivorship outcomes were comparable between both cohorts at 5-year follow-up.

Our study is the first large, single centre, prospective series at 5 years follow-up to report the clinical and radiological results of the Attune® system. This study was performed in a high-volume arthroplasty centre comparing our two most commonly used implants. All patients were followed up prospectively by the hospital's joint registry and we used the MKSRES tool for the analysis of all radiographs. This study adds to several, shorter-term follow-up studies that analyse the presence and significance of RLLs, specifically for the Attune® system. Concern has been raised about the early presence of radiolucent lines and early failure of the Attune® as a result of tibial tray de-bonding at the I/C interface [4, 16, 30]. The results from our study are consistent with a recent meta-analysis of 3,861 original, cemented Attune® TKAs which demonstrated low rates of aseptic loosening (1.2%) and revision surgery (0.9%) despite a 21.4% incidence of RLLs [21]. Our suggestion is that RLLs may not always be clinically significant, although we advise close clinical follow-up of these patients to monitor how this system performs long-term.

Interestingly, Ranawat et al. and Hamilton et al. both found no difference between the presence of RLLs and survivorship between the Attune^®^ and its predecessor the PFC Sigma^®^ system after 24 months follow-up [16, 22]. Furthermore, Robinson et al. found no difference in the rates of RLL or tibial debonding between the Attune^®^ and a matched cohort of patients with PFC Sigma^®^/Vanguard^®^ implants at 2 years [26].

Similar to our results, Giaretta et al. found an increased incidence of RLLs using the Attune^®^ but found that this had no correlation with rates of aseptic loosening. The clinical significance of RLLs and their association with aseptic loosening remains controversial which has led to a more detailed approach when analysing their presence. It is suggested that pathological radiolucencies i.e. those which are abnormal and typically associated with infection or aseptic loosening are progressive, poorly-defined and over 2 mm thick. In contrast, physiological radiolucencies (i.e. a normal finding) develop within the first post-operative year, become stable thereafter and are no more than 2 mm [14]. There was no difference in the incidence of progressive or stable lines between the Attune^®^ and Triathlon^®^ in our cohorts.

In our study, there was no statistically significant difference in revision-free survival between the Attune^®^ and Triathlon^®^ systems at 5 years (97.8% vs. 95.8% respectively). Furthermore, both devices demonstrated survivorship that is consistent with large joint database registries and cohort studies at a similar period of follow-up. The current 5-year cumulative revision rates reported by the Australian Orthopaedic Association National Joint Registry (AOANJR) are; Attune^®^ CR 3.1%, PS 2.6%; Triathlon^®^ CR 2.4%, PS 3.8% [23]. This compares to the data published by the New Zealand Joint Registry; Attune^®^ FB 2.06%, RP 1.37%; Triathlon^®^ 1.98% [25]. Hamilton et al. reported no difference between the survival rates for the Attune^®^ and PFC^®^ systems at 3 years follow-up. In support of our findings, they also noted that both implants had similar indications for revision surgery [15].

With regards to reasons for aseptic loosening, the appropriate cementation technique remains a controversial and a heavily-debated topic. Contributing factors to successful cementation include; ensuring complete cementation using 40g of cement for the tibial tray, adequately timing the cement application, using pulsed lavage followed by thorough drying to eliminate marrow infiltration and surgeon experience [3, 27, 28]. Both prostheses used in our study were implanted using the same cement by arthroplasty-trained surgeons.

Often, the design features of the tibial baseplate are highlighted as potential causes of early aseptic failure [4]. A comparative retrieval study from Cerquiglini et al. demonstrated that the failure mechanism of the Attune^®^ tibial tray is different to other implants. They observed no evidence of cement attached to the underside of the tibial tray on any of the 1st generation Attune^®^ protheses in their study. The authors attributed this to the lack of separate cement pockets seen in previous design models from the same manufacturer that were also tested [6].

In 2017, the manufacturer launched the redesigned Attune^®^ S + tibial tray with additional cement pockets, an under cut and increased surface roughness. Concerns over the early aseptic failure of modern TKAs is not an issue unique to the Attune^®^ prostheses. Keohane et al. recently reported an exceptionally high rate of aseptic loosening in the NexGen^®^ implant design in their series which has subsequently been recalled by the manufacturer [18].

Our data suggests improved outcomes in terms of knee specific and general quality of life outcomes when the Attune^®^ is compared to the Triathlon^®^. This was demonstrated by modest improvements in the OKS and EQ-5D scores at 5 years post-operatively. Of note, these differences did not reach the established minimum clinically important differences for these measures. Importantly, we found the presence of RLLs at the tibial component of the Attune^®^ system did not influence patient reported outcome measure data.

This is the first time that these two commonly used implants have been directly compared in a similar patient cohort. However, the satisfactory PROMs associated with the Attune^®^ system have been reported previously with improvements on its predecessor and excellent results at 1 and 2 years follow-up [8, 16, 30, 34]. Willburger & Oberberg also report a trend in the Attune^®^ towards less post-operative pain and better clinical results compared to the PFC Sigma^®^ at 5 years follow-up [35].

The main limitation of our study was that there were several surgeons using each implant which allowed for variation in the procedures. This data also represents the experience of one hospital and may not be generalisable to all patients. Additionally, tangential images are important to carefully identify RLLs, thus malposition of the patient during imaging might not allow for accurate review to detect all RLLs. We did not control between the different levels of constraint for each implant system in this study. All Attune^®^ prostheses used in this study were the original tibial tray design and therefore results are not applicable to the new Attune^®^ S+ tray.

## Conclusion

The Attune^®^ system showed an increased incidence of RLLs at the tibial tray in comparison with the Triathlon^®^ system. There was no difference in the overall incidence of RLLs observed between both systems. Interestingly, this did not lead to an increase in all-cause revision rates or revision for aseptic loosening at 5-years follow-up. Patient satisfaction and overall quality of life favoured the Attune^®^ system at 5 years follow-up. Both implants were associated with excellent midterm survivorship and high patient satisfaction.

## Data Availability

Not applicable.

## Abbreviations

TKA: Total knee arthroplasty
RLLs: Radiolucent lines
INOR: Irish National Orthopaedic Registry
C/B: Cement-bone
I/C: Implant-cement
AP: Anteroposterior
OKS: Oxford Knee Score
MCID: Minimal clinically important difference
PROM: Patient reported outcome measure
SD: Standard deviation
CR: Cruciate retaining
PS: Posterior stabilised
FB: Fixed bearing
RP: Rotating platform
MKSRES: Modern Knee Society Radiographic Evaluation System
